# Molecular data highlight hybridization in squirrel monkeys (*Saimiri*, Cebidae)

**DOI:** 10.1590/1678-4685-GMB-2016-0091

**Published:** 2016-10-31

**Authors:** Jeferson Carneiro, Luis Fernando da Silva Rodrigues-Filho, Horacio Schneider, Iracilda Sampaio

**Affiliations:** 1 Universidade Federal do Pará, Campus Universitário de Bragança, PA, Brazil.

**Keywords:** Saimiri, squirrel monkeys, Alu elements, hybridization

## Abstract

Hybridization has been reported increasingly frequently in recent years, fueling the debate on its role in the evolutionary history of species. Some studies have shown that hybridization is very common in captive New World primates, and hybrid offspring have phenotypes and physiological responses distinct from those of the "pure" parents, due to gene introgression. Here we used the TA15 *Alu* insertion to investigate hybridization in the genus *Saimiri*. Our results indicate the hybridization of *Saimiri boliviensis peruviensis* with *S. sciureus macrodon*, and *S. b. boliviensis* with *S. ustus*. Unexpectedly, some hybrids of both *S. boliviensis peruviensis* and *S. b. boliviensis* were homozygous for the absence of the insertion, which indicates that the hybrids were fertile.

The Neotropical squirrel monkey genus *Saimiri* is one of the many platyrrhine taxa subject to controversy and uncertainty in terms of its species diversity and phylogenetic relationships (Alfaro *et al.*, 2015). This genus is distributed primarily in the Amazon basin and Guianas (Figure 1), except *Saimiri oerstedii*, which is found in Central America, Costa Rica and Panama (Chiou *et al.*, 2011). One of the most recent biogeographical studies of *Saimiri* (Alfaro *et al.*, 2015) indicated that the species diversity of the genus is the product of a recent pan-Amazonian radiation. Based on the 14 clades identified in the analysis of the mitochondrial DNA (D-loop and cytb), these authors suggested a provisional taxonomy consisting of *S. sciureus*, *S. oerstedii* (*S. o. oerstedii* and *S. o. citronellus*), *S. collinsi*, *S. ustus* (A, B, and C lineages), *S. boliviensis, S. cassiquiarensis* (*S. c. cassiquiarensis, S. c. albigena, S. c. macrodon* A, *S. c. macrodon* B, and *S. c. macrodon* C), and *S. vanzolinii*. Analyzing mitochondrial DNA (CoxI and CoxII), Ruiz-García *et al.* (2015) proposed the following classification: *S. oerstedii*, with two subspecies (*S. o. oerstedii* and *S. o. citrinellus*), *S. vanzolinii* and *S. sciureus*, with two subspecies, *S. s. boliviensis* [with two lineages: 1 (*boliviensis*) and 2 (*peruviensis*)] and *S. s. sciureus* [with 12 lineages: 1 (*sciureus*), 2(*cassiquiarensis*), 3(*ustus* I=A), 4(*ustus* II = B), 5(*ustus* III = C), 6 (*macrodon* I=D),7(*macrodon* II = E), 8 (*macrodon* III = F), 9 (*macrodon* IV = G), 10 (*macrodon* V = H), 11 (*collinsi*) and 12 (*albigena*)].

**Figure 1:**
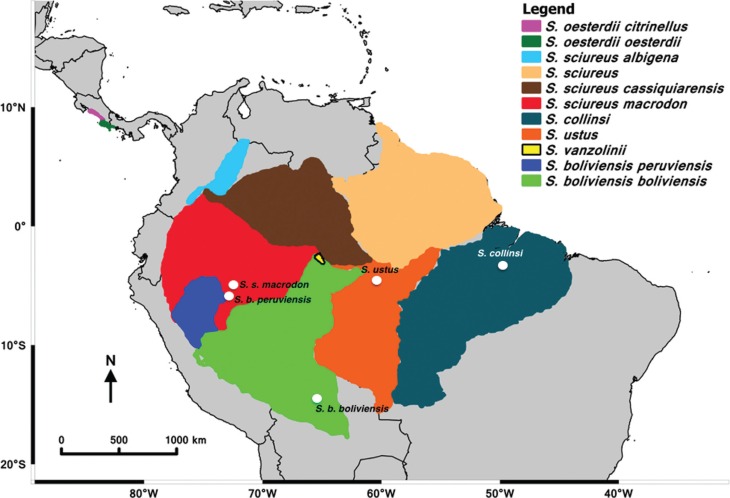
Map showing the geographical distributions of *Saimiri* species and the original sites where each one of the five populations studied was found. The majority of *S. b. boliviensis* specimens where captured in Santa Cruz de La Sierra, Bolivia (region in green) and later transported to CAPRIM in Argentina. Those from *S. b. peruviensis* (region in blue) and *S. sciureus macrodon* species (region in red) where captured in the vicinity of Iquitos, Peru, and then transported to CRCP/IVITA in Iquitos. *S. ustus* (region in orange) and *S. collinsi* specimens (region in aquamarine blue) were captured and sampled in the forest.

*Saimiri* populations occupy ample geographic areas (Figure 1), with many potential zones of contact that provide opportunities for hybridization between neighboring taxa (Hershkovitz, 1984). Thorington Jr (1985) reported cases of hybridization between *S. ustus* and *S. sciureus* on the east bank of the Tapajós River. Silva *et al.* (1992) investigated 49 specimens from a region in Peru occupied by both *S. b. peruviensis* and *S. s. macrodon*. By analyzing biochemical markers, these authors found clear evidence of admixture in approximately 45% of the individuals. Costello *et al.* (1993) also reported hybrids between *S. ustus* and *S. sciureus* from a region between the Madeira and Tapajós rivers.

Natural hybridization is the subject of a great deal of debate due to its potential importance as an evolutionary mechanism, especially for speciation, in addition to its relevance for taxonomy, conservation and species extinction (Mallet, 2005, 2007; Genovart, 2009;). Hybridization is known to have played a role in the evolutionary history of at least one quarter of plants and 10% of animal species (Rieseberg, 1997; Seehausen, 2004). Arnold and Meyer (2006) concluded that reticulate evolution is a common process in the evolutionary history of animals, with numerous examples of the formation of new taxa as a consequence of introgressive hybridization. In primates, this phenomenon has been reported in both captivity and the natural environment (Schreiber *et al.*, 1998; Zinner *et al.*, 2009; Matauschek *et al.*, 2011). However, the exact role of hybridization in the evolutionary history of an organism is usually unclear, and reticulate evolution represents a potential pitfall for phylogenetic reconstructions. Arnold and Meyer (2006) suggested that the accuracy of some phylogenetic constructs of New World monkeys is probably weakened by hybridization events that occurred in the past. While it is difficult to detect hybridization events, Osterholz *et al.* (2008) described the integration of an *Alu* element in *S. boliviensis*, which is absent in *S. sciureus*.

In the human genome, *Alu* elements are the most abundant transposable features (Kriegs *et al.*, 2007), and these elements are now known to comprise approximately 10% of the primate genome (Batzer and Deininger, 2002; Zhang *et al.*, 2002). Once inserted into the genome of a species during its evolutionary history, *Alu* insertions will be present in all the descendants of that species. An *Alu* insertion is thus a single and irreversible event (Hamdi *et al.*, 1999; Shedlock and Okada, 2000; Salem *et al.*, 2003), and represents a marker free of homoplasies. The present study investigated the potential occurrence of hybridization in free-living populations of *S. boliviensis*, based on the presence or absence of *Alu*TA15, as described by Osterholz *et al.* (2008).

We examined 107 samples of *Saimiri*: two *S. sciureus macrodon*, 16 *S. collinsi*, 17 *S. ustus*, 22 *S. boliviensis peruviensis* and 50 *S. b. boliviensis* (Table 1)*.* All the individuals sampled were born in the wild, although in some cases, the blood samples were collected in captivity. The samples of *S. collinsi* were collected from animals captured during the rescue operation of the UHE Tucurui hydroelectric reservoir in Para, Brazil (La Rovere and Mendes, 2000), and those of *S. ustus* at UHE Samuel, in Rondonia (Fearnside, 2005). The samples of *S. b. boliviensis*, *S. b. peruviensis* and *S. s. macrodon* were obtained from two captive facilities, the "Centro de Reproducción y Conservación de Primates No Humanos" (CRCP/IVITA) in Iquitos, Peru, and the "Centro Argentinode Primates" (CAPRIM) in Corrientes, Argentina. The species were identified based on the morphological characteristics described by Hershkovitz (1984). *S. b. boliviensis* has a white zone around the eyes exhibiting sparse white hairs and a flattened arch over the eyes (roman arch) while in *S. s macrodon* the arch formed above each eye is more evident and has been named as a "gothic arch".

**Table 1:** Species, specimen code, locality, geographical coordinates and origin of the specimens analyzed in the present study.

Taxa	Code	Locality	Coordinates		Origin
*Saimiri boliviensis boliviensis*	SBB 2103	Santa Cruz de La Sierra, Bolivia	17°20'	64°03'	CAPRIM
*Saimiri boliviensis boliviensis*	SBB 2104	Santa Cruz de La Sierra, Bolivia	17°20'	64°03'	CAPRIM
*Saimiri boliviensis boliviensis*	SBB 2105	Santa Cruz de La Sierra, Bolivia	17°20'	64°03'	CAPRIM
*Saimiri boliviensis boliviensis*	SBB 2111	Santa Cruz de La Sierra, Bolivia	17°20'	64°03'	CAPRIM
*Saimiri boliviensis boliviensis*	SBB 2112	Santa Cruz de La Sierra, Bolivia	17°20'	64°03'	CAPRIM
*Saimiri boliviensis boliviensis*	SBB 2113	Santa Cruz de La Sierra, Bolivia	17°20'	64°03'	CAPRIM
*Saimiri boliviensis boliviensis*	SBB 2114	Santa Cruz de La Sierra, Bolivia	17°20'	64°03'	CAPRIM
*Saimiri boliviensis boliviensis*	SBB 2115	Santa Cruz de La Sierra, Bolivia	17°20'	64°03'	CAPRIM
*Saimiri boliviensis boliviensis*	SBB 2116	Santa Cruz de La Sierra, Bolivia	17°20'	64°03'	CAPRIM
*Saimiri boliviensis boliviensis*	SBB 2118	Santa Cruz de La Sierra, Bolivia	17°20'	64°03'	CAPRIM
*Saimiri boliviensis boliviensis*	SBB 2119	Santa Cruz de La Sierra, Bolivia	17°20'	64°03'	CAPRIM
*Saimiri boliviensis boliviensis*	SBB 2120	Santa Cruz de La Sierra, Bolivia	17°20'	64°03'	CAPRIM
*Saimiri boliviensis boliviensis*	SBB 2121	Santa Cruz de La Sierra, Bolivia	17°20'	64°03'	CAPRIM
*Saimiri boliviensis boliviensis*	SBB 2123	Santa Cruz de La Sierra, Bolivia	17°20'	64°03'	CAPRIM
*Saimiri boliviensis boliviensis*	SBB 2124	Santa Cruz de La Sierra, Bolivia	17°20'	64°03'	CAPRIM
*Saimiri boliviensis boliviensis*	SBB 2126	Santa Cruz de La Sierra, Bolivia	17°20'	64°03'	CAPRIM
*Saimiri boliviensis boliviensis*	SBB 2128	Santa Cruz de La Sierra, Bolivia	17°20'	64°03'	CAPRIM
*Saimiri boliviensis boliviensis*	SBB 2129	Santa Cruz de La Sierra, Bolivia	17°20'	64°03'	CAPRIM
*Saimiri boliviensis boliviensis*	SBB 2130	Santa Cruz de La Sierra, Bolivia	17°20'	64°03'	CAPRIM
*Saimiri boliviensis boliviensis*	SBB 2131	Santa Cruz de La Sierra, Bolivia	17°20'	64°03'	CAPRIM
*Saimiri boliviensis boliviensis*	SBB 2132	Santa Cruz de La Sierra, Bolivia	17°20'	64°03'	CAPRIM
*Saimiri boliviensis boliviensis*	SBB 2133	Santa Cruz de La Sierra, Bolivia	17°20'	64°03'	CAPRIM
*Saimiri boliviensis boliviensis*	SBB 2134	Santa Cruz de La Sierra, Bolivia	17°20'	64°03'	CAPRIM
*Saimiri boliviensis boliviensis*	SBB 2135	Santa Cruz de La Sierra, Bolivia	17°20'	64°03'	CAPRIM
*Saimiri boliviensis boliviensis*	SBB 2136	Santa Cruz de La Sierra, Bolivia	17°20'	64°03'	CAPRIM
*Saimiri boliviensis boliviensis*	SBB 2140	Santa Cruz de La Sierra, Bolivia	17°20'	64°03'	CAPRIM
*Saimiri boliviensis boliviensis*	SBB 2141	Santa Cruz de La Sierra, Bolivia	17°20'	64°03'	CAPRIM
*Saimiri boliviensis boliviensis*	SBB 2142	Santa Cruz de La Sierra, Bolivia	17°20'	64°03'	CAPRIM
*Saimiri boliviensis boliviensis*	SBB 2143	Santa Cruz de La Sierra, Bolivia	17°20'	64°03'	CAPRIM
*Saimiri boliviensis boliviensis*	SBB 2144	Santa Cruz de La Sierra, Bolivia	17°20'	64°03'	CAPRIM
*Saimiri boliviensis boliviensis*	SBB 2145	Santa Cruz de La Sierra, Bolivia	17°20'	64°03'	CAPRIM
*Saimiri boliviensis boliviensis*	SBB 2146	Santa Cruz de La Sierra, Bolivia	17°20'	64°03'	CAPRIM
*Saimiri boliviensis boliviensis*	SBB 2149	Santa Cruz de La Sierra, Bolivia	17°20'	64°03'	CAPRIM
*Saimiri boliviensis boliviensis*	SBB 2150	Santa Cruz de La Sierra, Bolivia	17°20'	64°03'	CAPRIM
*Saimiri boliviensis boliviensis*	SBB 2151	Santa Cruz de La Sierra, Bolivia	17°20'	64°03'	CAPRIM
*Saimiri boliviensis boliviensis*	SBB 2153	Santa Cruz de La Sierra, Bolivia	17°20'	64°03'	CAPRIM
*Saimiri boliviensis boliviensis*	SBB 2157	Santa Cruz de La Sierra, Bolivia	17°20'	64°03'	CAPRIM
*Saimiri boliviensis boliviensis*	SBB 2158	Santa Cruz de La Sierra, Bolivia	17°20'	64°03'	CAPRIM
*Saimiri boliviensis boliviensis*	SBB 2159	Santa Cruz de La Sierra, Bolivia	17°20'	64°03'	CAPRIM
*Saimiri boliviensis boliviensis*	SBB 2160	Santa Cruz de La Sierra, Bolivia	17°20'	64°03'	CAPRIM
*Saimiri boliviensis boliviensis*	SBB 2164	Santa Cruz de La Sierra, Bolivia	17°20'	64°03'	CAPRIM
*Saimiri boliviensis boliviensis*	SBB 2165	Santa Cruz de La Sierra, Bolivia	17°20'	64°03'	CAPRIM
*Saimiri boliviensis boliviensis*	SBB 2168	Santa Cruz de La Sierra, Bolivia	17°20'	64°03'	CAPRIM
*Saimiri boliviensis boliviensis*	SBB 2169	Santa Cruz de La Sierra, Bolivia	17°20'	64°03'	CAPRIM
*Saimiri boliviensis boliviensis*	SBB 2170	Santa Cruz de La Sierra, Bolivia	17°20'	64°03'	CAPRIM
*Saimiri boliviensis boliviensis*	SBB 2171	Santa Cruz de La Sierra, Bolivia	17°20'	64°03'	CAPRIM
*Saimiri boliviensis boliviensis*	SBB 2174	Santa Cruz de La Sierra, Bolivia	17°20'	64°03'	CAPRIM
*Saimiri boliviensis boliviensis*	SBB 2176	Santa Cruz de La Sierra, Bolivia	17°20'	64°03'	CAPRIM
*Saimiri boliviensis boliviensis*	SBB 21?5	Santa Cruz de La Sierra, Bolivia	17°20'	64°03'	CAPRIM
*Saimiri boliviensis boliviensis*	SBB 21??	Santa Cruz de La Sierra, Bolivia	17°20'	64°03'	CAPRIM
*Saimiri boliviensis peruviensis*	SBP 1893	East bank of the Marañón River, Peru	06°41'	76°08'	CRCP/IVITA
*Saimiri boliviensis peruviensis*	SBP 1906	East bank of the Marañón River, Peru	06°41'	76°08'	CRCP/IVITA
*Saimiri boliviensis peruviensis*	SBP 1908	East bank of the Marañón River, Peru	06°41'	76°08'	CRCP/IVITA
*Saimiri boliviensis peruviensis*	SBP 1915	East bank of the Marañón River, Peru	06°41'	76°08'	CRCP/IVITA
*Saimiri boliviensis peruviensis*	SBP 1916	East bank of the Marañón River, Peru	06°41'	76°08'	CRCP/IVITA
*Saimiri boliviensis peruviensis*	SBP 1917	East bank of the Marañón River, Peru	06°41'	76°08'	CRCP/IVITA
*Saimiri boliviensis peruviensis*	SBP 1918	East bank of the Marañón River, Peru	06°41'	76°08'	CRCP/IVITA
*Saimiri boliviensis peruviensis*	SBP 1919	East bank of the Marañón River, Peru	06°41'	76°08'	CRCP/IVITA
*Saimiri boliviensis peruviensis*	SBP 1920	East bank of the Marañón River, Peru	06°41'	76°08'	CRCP/IVITA
*Saimiri boliviensis peruviensis*	SBP 1922	East bank of the Marañón River, Peru	06°41'	76°08'	CRCP/IVITA
*Saimiri boliviensis peruviensis*	SBP 1923	East bank of the Marañón River, Peru	06°41'	76°08'	CRCP/IVITA
*Saimiri boliviensis peruviensis*	SBP 1925	East bank of the Marañón River, Peru	06°41'	76°08'	CRCP/IVITA
*Saimiri boliviensis peruviensis*	SBP 1926	East bank of the Marañón River, Peru	06°41'	76°08'	CRCP/IVITA
*Saimiri boliviensis peruviensis*	SBP 1929	East bank of the Maranon river, Peru	06°41'	76°08'	CRCP/IVITA
*Saimiri boliviensis peruviensis*	SBP 1931	East bank of the Maranon river, Peru	06°41'	76°08'	CRCP/IVITA
*Saimiri boliviensis peruviensis*	SBP 1932	East bank of the Marañón River, Peru	06°41'	76°08'	CRCP/IVITA
*Saimiri boliviensis peruviensis*	SBP 1933	East bank of the Marañón River, Peru	06°41'	76°08'	CRCP/IVITA
*Saimiri boliviensis peruviensis*	SBP 1934	East bank of the Marañón River, Peru	06°41'	76°08'	CRCP/IVITA
*Saimiri boliviensis peruviensis*	SBP 1936	East bank of the Maranon river, Peru	06°41'	76°08'	CRCP/IVITA
*Saimiri boliviensis peruviensis*	SBP 1937	East bank of the Maranon river, Peru	06°41'	76°08'	CRCP/IVITA
*Saimiri boliviensis peruviensis*	SBP 1939	East bank of the Marañón River, Peru	06°41'	76°08'	CRCP/IVITA
*Saimiri boliviensis peruviensis*	SBP 1941	East bank of the Marañón River, Peru	06°41'	76°08'	CRCP/IVITA
*Saimiri sciureus macrodon*	SSM 1945	Ucayali River (Caserio Bagazan, Quebrada Carahuayte), Peru	07°52'	74°34'	CRCP/IVITA
*Saimiri sciureus macrodon*	SSM 1946	Ucayali River (Caserio Bagazan, Quebrada Carahuayte), Peru	07°52'	74°34'	CRCP/IVITA
*Saimiri collinsi*	SC 34	Left bank of the Tocantins River, Pará, Brazil	03°52'	49°42'	Free-living
*Saimiri collinsi*	SC 36	Left bank of the Tocantins River, Pará, Brazil	03°52'	49°42'	Free-living
*Saimiri collinsi*	SC 410	Left bank of the Tocantins River, Pará, Brazil	03°52'	49°42'	Free-living
*Saimiri collinsi*	SC 473	Left bank of the Tocantins River, Pará, Brazil	03°52'	49°42'	Free-living
*Saimiri collinsi*	SC 525	Left bank of the Tocantins River, Pará, Brazil	03°52'	49°42'	Free-living
*Saimiri collinsi*	SC 626	Left bank of the Tocantins River, Pará, Brazil	03°52'	49°42'	Free-living
*Saimiri collinsi*	SC 627	Left bank of the Tocantins River, Pará, Brazil	03°52'	49°42'	Free-living
*Saimiri collinsi*	SC 686	Left bank of the Tocantins River, Pará, Brazil	03°52'	49°42'	Free-living
*Saimiri collinsi*	SC 749	Left bank of the Tocantins River, Pará, Brazil	03°52'	49°42'	Free-living
*Saimiri collinsi*	SC 847	Left bank of the Tocantins River, Pará, Brazil	03°52'	49°42'	Free-living
*Saimiri collinsi*	SC 863	Left bank of the Tocantins River, Pará, Brazil	03°52'	49°42'	Free-living
*Saimiri collinsi*	SC 865	Left bank of the Tocantins River, Pará, Brazil	03°52'	49°42'	Free-living
*Saimiri collinsi*	SC 873	Left bank of the Tocantins River, Pará, Brazil	03°52'	49°42'	Free-living
*Saimiri collinsi*	SC 1502	Left bank of the Tocantins River, Pará, Brazil	03°52'	49°42'	Free-living
*Saimiri collinsi*	SC 1549	Left bank of the Tocantins River, Pará, Brazil	03°52'	49°42'	Free-living
*Saimiri collinsi*	SC 1679	Left bank of the Tocantins River, Pará, Brazil	03°52'	49°42'	Free-living
*Saimiri ustus*	SU 2257	Right bank of the Jamari River, Rondônia, Brazil	08°56	63°21'	Free-living
*Saimiri ustus*	SU 2305	Right bank of the Jamari River, Rondônia, Brazil	08°56	63°21'	Free-living
*Saimiri ustus*	SU 2354	Right bank of the Jamari River, Rondônia, Brazil	08°56	63°21'	Free-living
*Saimiri ustus*	SU 2450	Right bank of the Jamari River, Rondônia, Brazil	08°56	63°21'	Free-living
*Saimiri ustus*	SU 2454	Right bank of the Jamari River, Rondônia, Brazil	08°56	63°21'	Free-living
*Saimiri ustus*	SU 2577	Right bank of the Jamari River, Rondônia, Brazil	08°56	63°21'	Free-living
*Saimiri ustus*	SU 3193	Right bank of the Jamari River, Rondônia, Brazil	08°56	63°21'	Free-living
*Saimiri ustus*	SU 4030	Right bank of the Jamari River, Rondônia, Brazil	08°56	63°21'	Free-living
*Saimiri ustus*	SU 4031	Left bank of the Jamari River, Rondônia, Brazil	08°52	63°15'	Free-living
*Saimiri ustus*	SU 4032	Left bank of the Jamari River, Rondônia, Brazil	08°52	63°15'	Free-living
*Saimiri ustus*	SU 4033	Left bank of the Jamari River, Rondônia, Brazil	08°52	63°15'	Free-living
*Saimiri ustus*	SU 4041	Left bank of the Jamari River, Rondônia, Brazil	08°52	63°15'	Free-living
*Saimiri ustus*	SU 4257	Left bank of the Jamari River, Rondônia, Brazil	08°52	63°15'	Free-living
*Saimiri ustus*	SU 4441	Left bank of the Jamari River, Rondônia, Brazil	08°52	63°15'	Free-living
*Saimiri ustus*	SU 4508	Left bank of the Jamari River, Rondônia, Brazil	08°52	63°15'	Free-living
*Saimiri ustus*	SU 4550	Left bank of the Jamari River, Rondônia, Brazil	08°52	63°15'	Free-living
*Saimiri ustus*	SU 4577	Left bank of the Jamari River, Rondônia, Brazil	08°52	63°15'	Free-living

UHE Tucurui= Tocantins River; UHE Samuel= Jamari River.

While *S. b. boliviensis* and *S. b. peruviensis* have an arch that is less pronounced over the eyes (roman arch), *S. b. peruviensis* has a crown pattern on the head which is less eumelanized than that of *S. b. boliviensis*. The specimens held at CRCP/IVITA were classified as *S. boliviensis peruviensis* (roman arch) and those from the vicinity of Iquitos (Figure 1) as *S. sciureus macrodon* (gothic arch), while the animals at CAPRIM, captured in Santa Cruz de La Sierra, Bolivia, were all *S. boliviensis boliviensis* (roman arch). Some of the animals at CAPRIM were born in captivity. Further details on the specimens and the geographical distribution of each population are presented in Table 1 and Figure 1. The material analyzed in the present study was part of the sample bank maintained by the Molecular Phylogenetics Laboratory at the Bragança campus of the Federal University of Para.

The total DNA was extracted using the Wizard Genomic kit (Promega, Madison, WI, USA) following the manufacturer's recommendations. The region of interest (*Alu*TA15) was amplified using the primers and the protocol described by Osterholz *et al.* (2008). The initial denaturation step was 2 min at 94 °C, followed by 40 cycles of denaturation (1 min at 94 °C), annealing (1 min at 58 °C), and extension (1 min at 72 °C), with a final extension step of 5 min at 72 °C. After amplification, the PCR products were separated electrophoretically in a 2% agarose gel at 60 V, 150 mA for 60 min together with a 1 kb plus DNA ladder (Invitrogen, Carsbad, CA, USA). All the fragments were stained with GelRed, as recommended by the manufacturer (Biotium, Hayward, CA, USA). Sequence reactions were conducted with a Big Dye v.3.1 kit (ABI BigDye® Terminator Mix; Applied Biosystems, Carlsbad, CA, USA), conducted in an ABI 3500xL sequencer (Applied Biosystems), to confirm that the region amplified by PCR was the fragment of interest (*Alu*TA15). The sequences were aligned and edited manually in the BioEdit program (Hall, 1999).

The primers designed by Osterholz *et al.* (2008) amplify fragments of distinct sizes depending on the presence or absence of the *Alu* insertion (*Alu*TA15). When the *Alu*TA15 insertion is present, a fragment of approximately 750 base pairs (bps) is generated, but when it is absent, a fragment of only 450 bps is generated. As the insertion is only present in *S. boliviensis* (Figure 2), in hybrids between this species and other *Saimiri* species, two fragments will be amplified, one with 750 bps and another with 450 bps.

**Figure 2:**
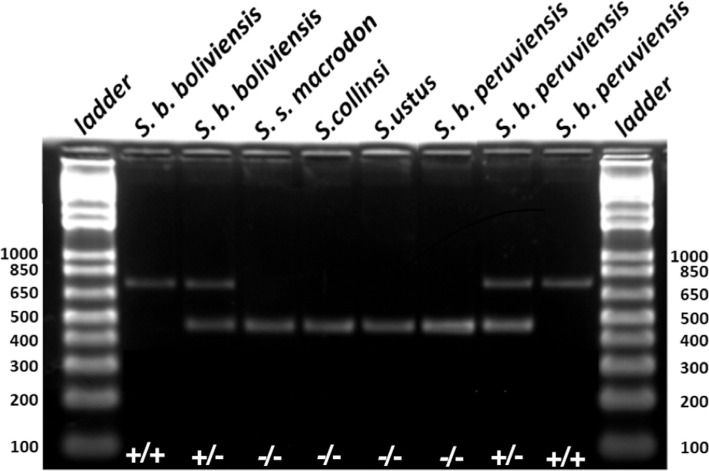
Electrophoresis gel showing the distribution of the three *Alu* genotypes (+/+; +/-; and -/-) in the five subspecies sampled in the present study. A 1 kb ladder placed at both sides of the gel indicates the size in base pairs (bp) of the two amplified fragments.

The *Alu*TA15 insertion was not detected in any of the individuals identified as *S. ustus* (n=17) from Rondonia, *S. collinsi* (n=16) from Para or *S. sciureus macrodon* (n=2) from Peru. All 35 individuals presented only one band of approximately 450 bps (Table 2). By contrast, 50 specimens from Santa Cruz de La Sierra, Bolivia, identified as *S. b. boliviensis*, presented the insertion, of which 90% were homozygous (+/+) and 10% (five individuals) were heterozygous (+/-) showing both bands (750 bps and 450 bps). This configuration was unexpected because Osterholz *et al.* (2008) proposed that the *Alu*TA15 element was inserted into the lineage that originated the extant species *S. boliviensis*, which implies that all *S. boliviensis* should be homozygous for *Alu*TA15 (+/+). Interestingly, all three possible combinations were found in the population previously identified as *S. b. peruviensis* from Peru (CRCP), with six individuals (28%) being homozygous for the insertion (+/+), eight (36%) being homozygous for its absence (-/-), and the other eight being heterozygous (+/-), showing both bands (750/450 bps) in the gel (Figure 2). Osterholz *et al.* (2008) also found three possible patterns of bands (+/+; +/-; -/-) for specimens that were previously identified as *S. b. peruviensis*. So again, if the *Alu*TA15 was inserted into the ancestral lineage of *S. boliviensis*, as proposed by Osterholz *et al.* (2008), it is unclear how specimens of this species could lack the insertion (-/-).

**Table 2:** Presence (+) or absence (-) of the *Alu* TA15 insertion in the *Saimiri* specimens analyzed in the present study.

	Number (% of the total) of specimens:			
	Homozygous	Heterozygous	Homozygous	
	-/-	-/+	+/+	Total
*Saimiri boliviensis peruviensis*	8 (36%)	8 (36%)	6 (28%)	22
*Saimiri boliviensis boliviensis*	0	5 (10%)	45 (90%)	50
*Saimiri collinsi*	16 (100%)	0	0	16
*Saimiri sciureus macrodon*	2 (100%)	0	0	2
*Saimiri ustus*	17 (100%)	0	0	17
Total:	32	13	51	107

It is well known that *Alu* elements are replicated in a copy-and-paste way in the primate genome, and once inserted into a genome, they cannot be excised. Given this, individuals phenotypically typical of *Saimiri b. peruviensis*, but heterozygous for the insertion (+/-), must be the result of natural hybridization, which would presumably have involved the geographically closest taxon, *S. sciureus macrodon*. Furthermore, the absence of the insertion (-/-) in morphologically typical *S. b. peruviensis* can only be accounted for by the crossing of hybrid (+/-) *Saimiri b. peruviensis* or crosses between a hybrid and *S. sciureus macrodon* (-/-). These crosses would generate 25% or 50% of descendants without the insertion (-/-) and with dubious or intermediate morphological characteristics, which would represent conclusive evidence that hybridization between *S. boliviensis* and *Saimiri sciureus macrodon* produces fertile offspring. However, only 10% of the 50 *Saimiri b. boliviensis* specimens were heterozygous (+/-), and probably originated from crosses with *Saimiri ustus*, due to the proximity of the geographical distribution of these species (Figure 2).

It is interesting to note that *S. b. peruviensis* and *S. s. macrodon* occur sympatrically in the region between the Marañón and Tapiche rivers in the Peruvian Amazonia, whereas *S. b. boliviensis* is parapatric with *S. s. macrodon* and *S. ustus*, which are separated by the Juruá and Purus-Guaporé Rivers, respectively (Hershkovitz, 1984). However, these rivers do not constitute an effective geographic barrier to gene flow in lizards (Souza *et al.*, 2013), primates, and other organisms (Gascon *et al.*, 2000), which implies that there may be gene flow between the present-day ranges of the three *Saimiri* species, resulting in hybridization between *Saimiri boliviensis* and *Saimiri sciureus* or *S. ustus*, as suggested by previous authors (Hershkovitz, 1984; Thorington Jr, 1985; Silva *et al.*, 1992, 1993; Osterholz *et al.*, 2008) based on morphological data.

Using chromosomal data, Jones and Ma (1975) were able to distinguish between *S. b. peruviensis* and *S. s. macrodon* from the vicinity of Iquitos (Peru) and Leticia (Colombia), respectively. Both species revealed a diploid number of 2n=42, with 10 meta/submetacentric, 22 acrocentric and 10 telocentric chromosomes in *S. b. peruviensis,* and 10 meta/submetacentric, 20 acrocrentic, and 12 telocentric chromosomes in *S. s. macrodon*. A hybrid produced in the laboratory between a male from Iquitos and a female from Leticia showed 10 meta/submetacentric, 11 acrocentic and 11 telocentric chromosomes. Lau and Arrighi (1976) using chromosomal banding analyses detected two nonhomologous pericentric inversions in the telocentric group of chromosomes of a squirrel monkey, *Saimiri sciureus,* suggesting that this individual was an intersubspecific hybrid whose parents originated from different geographical locations. Recently, Ruiz-García *et al.* (2015) also found evidence of hybridization between *Saimiri* species based on mitochondrial markers (Cox1 and Cytb), emphasizing the importance of this process in the species-level diversification of this genus. In fact, these authors concluded that this genus comprises only three species, *S. oerstedi*, *S. sciureus*, and *Saimiri vanzolinii*, which diversified during the Pleistocene. This is consistent with the estimate of Alfaro *et al.* (2015), who concluded that *S. boliviensis* diverged from the other *Saimiri* species less than 1.5 Ma.

Hybridization may be a catalyst not only for speciation but also for major evolutionary innovations (Mallet (2007). Hybridization between *Saimiri* species appears to be common, and as Mallet (2007) concludes at page 182: "*most speciation involves natural selection; natural selection requires genetic variation; genetic variation is enhanced by hybridization; and hybridization and introgression between species is a regular occurrence, especially in rapidly radiating groups*". On the basis of the evidence presented here, this appears to have been the case in *Saimiri*.
